# Association between Stroke Risk and Influenza Vaccination in Patients with Gout: A Nationwide Population-Based Study

**DOI:** 10.3390/vaccines10081278

**Published:** 2022-08-08

**Authors:** Chun-Chao Chen, Kuan-Ting Chou, Ju-Chi Liu, Chun-Chih Chiu, Tsung-Yeh Yang, Cheng-Hsin Lin, Yu-Ann Fang, William Jian, Meng-Huan Lei, Hsien-Tang Yeh, Min-Huei Hsu, Wen-Rui Hao

**Affiliations:** 1 Division of Cardiology, Department of Internal Medicine, Shuang Ho Hospital, Taipei Medical University, New Taipei City 235, Taiwan; 2 Taipei Heart Institute, Taipei Medical University, Taipei 110, Taiwan; 3 Division of Cardiology, Department of Internal Medicine, School of Medicine, College of Medicine, Taipei Medical University, Taipei 110, Taiwan; 4 Graduate Institute of Medical Sciences, College of Medicine, Taipei Medical University, Taipei 110, Taiwan; 5 Department of Medical Education, Shuang Ho Hospital, Taipei Medical University, New Taipei City 235, Taiwan; 6 Division of Cardiovascular Surgery, Department of Surgery, Shuang Ho Hospital, Taipei Medical University, New Taipei City 235, Taiwan; 7 Division of Cardiovascular Surgery, Department of Surgery, School of Medicine, College of Medicine, Taipei Medical University, Taipei 110, Taiwan; 8 Department of Emergency, University Hospitals Cleveland Medical Center, Cleveland, OH 44106, USA; 9 Cardiovascular Center, Lo-Hsu Medical Foundation Luodong Poh-Ai Hospital, Yilan 265, Taiwan; 10 Department of Surgery, Lotung Poh-Ai Hospital, Luodong 265, Taiwan; 11 Graduate Institute of Data Science, College of Management, Taipei Medical University, Taipei 110, Taiwan; 12 Department of Neurosurgery, Wan-Fang Hospital, Taipei Medical University, Taipei 110, Taiwan

**Keywords:** influenza vaccine, gout, stroke

## Abstract

The risk of stroke in patients with gout is high. The effect of vaccines in lowering the stroke risk in patients with gout remains unclear. We retrospectively analyzed 23,949 patients with gout (age ≥ 55 years) from the National Health Insurance Research Database over a 12-year period. The patients were divided into vaccinated (n = 11,649) and unvaccinated groups (n = 12,300). Overall, the vaccinated group had significantly lower risks of all stroke, hemorrhagic stroke, and ischemic stroke than the unvaccinated group (adjusted hazard ratio [aHR], 0.59 and 95% confidence interval [CI], 0.55–0.63; aHR, 0.60 and 95% CI, 0.49–0.73; and aHR, 0.60 and 95% CI, 0.55–0.65, respectively). The association appeared to be dose-dependent for both hemorrhagic and ischemic stroke (hemorrhagic stroke: aHR, 0.81 and 95% CI, 0.61–1.08; aHR, 0.80 and 95% CI, 0.62–1.02; and aHR, 0.37 and 95% CI, 0.28–0.48; ischemic stroke: aHR, 0.83 and 95% CI, 0.74–0.94; aHR, 0.73 and 95% CI, 0.65–0.81; and aHR, 0.42 and 95% CI, 0.38–0.47 for patients vaccinated 1, 2 or 3, and ≥4 times, respectively, during the follow-up period). Patients with a history of atrial fibrillation did not have a lower risk of hemorrhagic stroke even after receiving four vaccinations (aHR, 0.59; 95% CI, 0.25–1.38). Influenza vaccination was associated with a lower risk of all stroke in people with gout, and the association appeared to be dose-dependent.

## 1. Introduction

Gout is the most common type of inflammatory arthritis in adults [1]. The prevalence of gout varies from 0.4% to 6.1% in most developed countries, and that in Taiwan was estimated to be 4.92% in 2004 [2]. The development of gout is predominantly caused by chronic elevation of serum uric acid (SUA) levels above the saturation point for monosodium urate crystal formation [2]. Uric acid is an end product of purine metabolism in humans. The SUA concentration was strongly correlated with risk factors for cardiovascular disease, such as body weight, serum glucose, and lipid levels [3]. In addition, the association between SUA concentration and stroke risk has been well documented. A recent meta-analysis of 13 cohort studies indicated that an elevated SUA level was significantly associated with a modestly increased risk of stroke [4].

Stroke is a major cause of death and disability worldwide [5]. A temporal relationship between influenza infection and stroke has been observed [6], with acute infection being associated with a transient increase in the risk of vascular events, including stroke [7]. Influenza vaccination was discovered to not only reduce the risk of influenza infection, but also to lower the risk of stroke [8]. The possible mechanism could be explained by the protective effect of influenza vaccination from influenza infection, which can induce a systemic inflammatory response and endothelial injury, and enhance thrombotic tendencies [9]. In a previous study, the neuroprotective effect from influenza vaccination among patients with high risk factors, such as diabetes and older age, has been demonstrated [8]. However, the possible positive effect of influenza vaccination on the risk of stroke in patients with gout remains unclear. The aim of the present study was to investigate the association between the risk of stroke and influenza vaccination in patients with gout from a nationwide population.

## 2. Materials and Methods

We used Taiwan’s National Health Insurance Research Database (NHIRD) to analyze the effect of influenza vaccination on stroke risk in patients with gout. The National Health Insurance (NHI) has been established since 1995, and the NHIRD has been described in detail in previous studies [10,11]. The NHIRD comprises the detailed health care data of >23 million enrollees, representing >99% of Taiwan’s entire population. The National Health Research Institutes further encrypt these data before researchers can access the database, although theoretically, NHIRD data alone are insufficient to identify any individuals. All researchers using the NHIRD and its data subsets must sign a written agreement declaring that they have no intention of obtaining information that could potentially violate the privacy of patients or care providers. Consequently, our data were delinked and deidentified. The present study was approved by the Joint Institutional Review Board of Taipei Medical University (approval no. N201804043).

**Figure 1:**
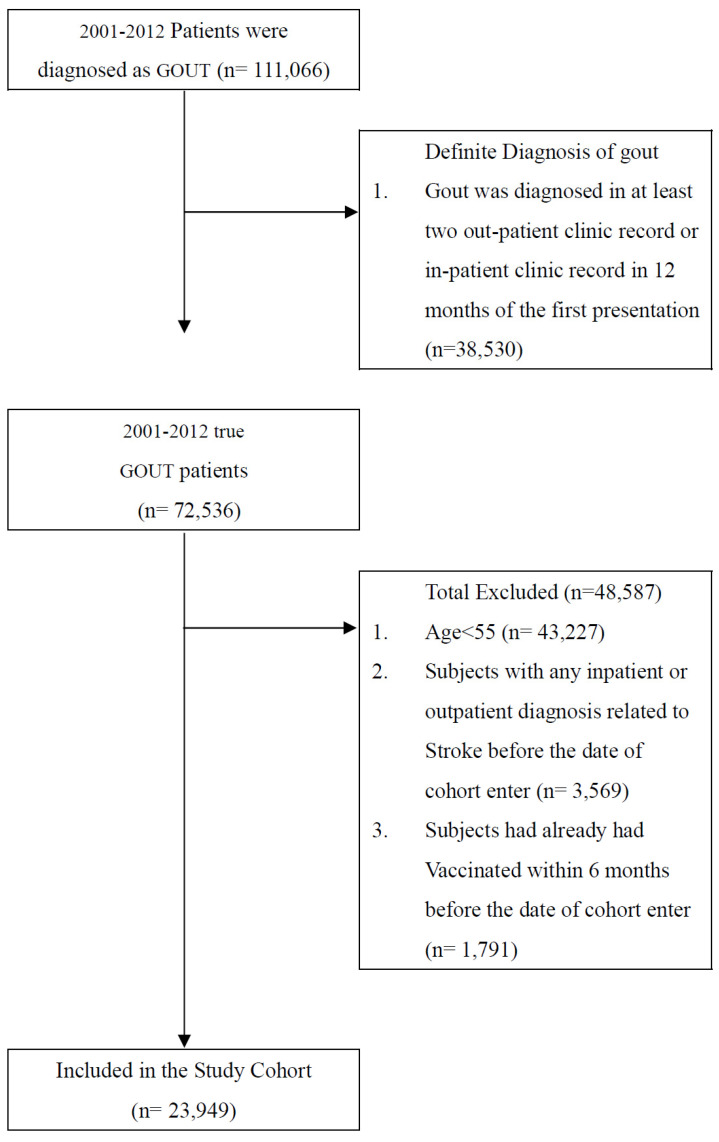
Data selection process.

We screened all patients who visited health care facilities in Taiwan with a diagnosis of gout (International Classification of Diseases, Ninth Revision, Clinical Modification [ICD-9-CM] code V77.5) between 1 January 2001 and 31 December 2012 (n = 111,066). Patients with no subsequent outpatient visit, emergency department visit, or inpatient hospitalization for gout within 1 year of the first presentation were excluded (n = 38,530) because they were considered to not have gout. An additional 48,587 patients were excluded because of the following: patients aged <55 years (n = 43,227), patients with any inpatient or outpatient diagnosis related to stroke before the cohort entry date (n = 3569), and patients who had been vaccinated within the 6 months before the cohort entry date (n = 1791). Finally, 23,949 patients were included in the study (Figure 1).

In Taiwan, influenza vaccination has been free for high-risk individuals (patients aged ≥50 years with type 2 diabetes, chronic liver infection, liver cirrhosis, cardiovascular disease, or chronic pulmonary disease) since 1998, and for all adults aged >65 years old since 2001. Vaccination status was identified by ICD-9-CM code V048 and/or the use of vaccine (confirmed by drug codes). Our primary endpoint was incidence of stroke (ICD-9-CM code V17.1) in patients with gout in all seasons during the follow-up years. The patients were followed until stroke diagnosis, withdrawal from the NHI, loss to follow-up, death, or as of 31 December 2012.

The potential confounders of this cohort study included sociodemographic characteristics (age, sex, urbanization level, and monthly income), comorbidities (Charlson comorbidity index, DM, hypertension, dyslipidemia, and atrial fibrillation (AF)), and medication use (allopurinol, benzbromarone, colchicine, aspirin, statin, renin-angiotensin antagonist (RAA), and metformin).

We used the propensity score method to reduce selection bias in the comparison between vaccinated and unvaccinated patients by accounting for covariates using a logistic regression model. The chi-square test was used for categorical variables, and the *t*-test was used for continuous variables. We also analyzed the association between influenza vaccination and stroke in patients with gout by using Cox proportional hazards regression. The influence of the dose effect of influenza vaccination on the incidence of stroke was also determined. Patients were categorized into four groups in accordance with their vaccination status (0, 1, 2, 3, or ≥4 vaccinations). These data were stratified in accordance with patients’ age, sex, comorbidity, and chronic medication use. A sensitivity analysis was performed to evaluate the differences and similarities between groups regarding influenza vaccination and risk of stroke. All statistical analyses were performed using SPSS 22.0 (SPSS Inc., Chicago, IL, USA) and SAS 9.4 software (SAS Institute Inc., Cary, NC, USA). Statistical significance was set at *p* < 0.05.

## 3. Results

### 3.1. Baseline Characteristics

Of the 23,949 eligible individuals, 11,649 (48.64%) received influenza vaccination, and the remaining 12,300 (51.35%) were unvaccinated. A significant difference was discovered between the two groups regarding their age distribution, level of urbanization, and monthly income. The prevalence of pre-existing medical comorbidities, including hypertension and AF, was higher in the vaccinated group; in contrast, the prevalence of dyslipidemia was higher in the unvaccinated group. Significant differences were also discovered with regard to comorbidity-associated medication use. Higher proportions of vaccinated patients than unvaccinated patients used allopurinol, benzbromarone, colchicine, aspirin, statin, RAA, and metformin for >28 days (Table 1).

### 3.2. Risk of Stroke in Vaccinated and Unvaccinated Patients in Accordance with Age and Sex

After adjustment for potential confounders, the incidence rate of all stroke was significantly lower in the vaccinated group than in the unvaccinated group (adjusted hazard ratio [aHR], 0.59; 95% CI, 0.55–0.63; *p* < 0.001; Table 2). Lower risks of hemorrhagic stroke (aHR, 0.60; 95% CI, 0.49–0.73; *p* < 0.001) and ischemic stroke (aHR, 0.60; 95% CI, 0.55–0.65; *p* < 0.001) were also discovered in the vaccinated group. Among the patients aged 55–64 years and 65–74 years, the risks of all stroke, hemorrhagic stroke, and ischemic stroke were significantly lower in the vaccinated group. The risk of hemorrhagic stroke was not significantly different between vaccinated and unvaccinated patients aged ≥75 years (aHR, 0.67; 95% CI, 0.44–1.02). In male patients, the risks of all stroke, hemorrhagic stroke, and ischemic stroke were significantly lower if they had been vaccinated. In female patients, the risks of all stroke and ischemic stroke were lower if they had been vaccinated, but the risk of hemorrhagic stroke was not significantly different between the vaccinated and unvaccinated patients (aHR, 0.76; 95% CI, 0.54–1.08).

### 3.3. Association between Number of Influenza Vaccinations and Risk Reduction of Hemorrhagic Stroke

In the main model, a significantly lower risk of hemorrhagic stroke was associated with a higher number of influenza vaccinations in patients with gout over the follow-up period (aHR, 0.81 and 95% CI, 0.61–1.08; aHR, 0.80 and 95% CI: 0.62–1.02; and aHR, 0.37 and 95% CI, 0.28–0.48 for patients vaccinated 1, 2, 3, and ≥4 times, respectively; Table 3). After an analysis of the additional covariates, a significantly lower risk of hemorrhagic stroke was determined in patients using different medications after they had received >4 vaccinations. In the subgroup analysis, patients with a history of DM, dyslipidemia, and hypertension were significantly protected from hemorrhagic stroke after receiving ≥4 vaccinations (aHR 0.33, 95% CI 0.19–0.58; aHR 0.28, 95% CI 0.16–0.51; and aHR 0.36, 95% CI 0.25–0.51, respectively). Patients with a history of AF did not exhibit a lower risk of hemorrhagic stroke even after receiving four vaccinations (aHR, 0.59; 95% CI, 0.25–1.38). The risk of hemorrhagic stroke was lower in patients without DM, dyslipidemia, hypertension, and AF after they had received ≥4 vaccinations (aHR, 0.37 and 95% CI, 0.27–0.51; aHR, 0.41 and 95% CI, 0.30–0.56; aHR, 0.38 and 95% CI, 0.25–0.60; and aHR, 0.35 and 95% CI, 0.26–0.47, respectively). The protective effect from hemorrhagic stroke was significant after a patient had received ≥4 vaccinations, regardless of which medication was used, except for metformin. Patients who used metformin for <28 days had lower risk of hemorrhagic stroke after receiving only one vaccination (aHR, 0.70 and 95% CI, 0.50–0.98; aHR, 0.74 and 95% CI, 0.55–0.99; and aHR, 0.36 and 95% CI, 0.26–0.50; *p* for trend <0.001 for patients vaccinated 1, 2 or 3, and ≥4 times, respectively).

### 3.4. Association between Number of Influenza Vaccinations and Reduction of Ischemic Stroke Risk

In the main model, the reduction of ischemic stroke risk was significant after the first vaccination, and a trend of risk reduction was observed as the number of vaccinations increased (aHR, 0.83 and 95% CI, 0.74–0.94; aHR, 0.73 and 95% CI, 0.65–0.81; and aHR, 0.42 and 95% CI, 0.38–0.47, all *p* < 0.001, for patients vaccinated 1, 2, 3, and ≥4 times, respectively). Similar findings were obtained for the main model with additional covariates and in most of the subgroup analyses. Patients older than 65 years and female patients benefited from vaccinations when two or three doses had been administered (aHR, 0.76 and 95% CI, 0.67–0.87; and aHR, 0.67 and 95% CI, 0.56–0.79 for patients aged >65 years and female patients, respectively). Patients with a history of DM had lower risk of developing ischemic stroke after two or three vaccination doses had been administered (aHR, 0.80; 95% CI, 0.66–0.96). Patients on medication for <28 days had a lower risk of developing ischemic stroke after two or three vaccination doses had been administered (aHR, 0.75 and 95% CI, 0.65–0.85; aHR, 0.78 and 95% CI, 0.67–0.90; aHR, 0.76 and 95% CI, 0.67–0.88; aHR, 0.68 and 95% CI, 0.54–0.86; aHR, 0.77 and 95% CI, 0.67–0.90; and aHR, 0.79 and 95% CI, 0.64–0.99 for allopurinol, benzbromarone, colchicine, aspirin, statin, and RAA, respectively). In contrast, patients on metformin for >28 days had a lower risk of developing ischemic stroke after two or three vaccination doses had been administered (aHR, 0.78; 95% CI, 0.65–0.95; Table 4).

## 4. Discussion

### 4.1. Main Findings

This nationwide population-based cohort study obtained several major findings. First, overall, people with gout had a lower risk of all stroke after receiving an influenza vaccination, but the reduction of hemorrhagic stroke risk was not highly significant in people with gout who were either female or aged >75 years. Second, the association between influenza vaccination and lower risk of stroke in patients with gout tended to be dose-dependent. The effect of reducing hemorrhagic stroke became apparent after four doses of the vaccine had been administered. In contrast, the effect of reducing ischemic stroke became apparent after one dose of the vaccine had been administered, and the trend in risk reduction became significant as the number of vaccinations increased. Third, regardless of comorbidities, patients with gout had a lower risk of hemorrhagic stroke after receiving more than four influenza vaccinations, except for those with gout and AF, in whom this effect was not discovered even after the patient had received more than four influenza vaccinations. In addition, regardless of comorbidities, patients with gout had lower risk of ischemic stroke after receiving one influenza vaccination, except if they had diabetes; these patients had a lower risk of ischemic stroke only after receiving two or three influenza vaccinations. Finally, among the patients with gout who had used metformin for <28 days, having received ≥1 vaccination was associated with a lower risk of hemorrhagic stroke. Those who had used metformin for >28 days required more than two or three vaccinations to obtain a protective effect against ischemic stroke. To the best of our knowledge, this is the first study to investigate the association between influenza vaccination and stroke risk in patients with gout.

### 4.2. Association between Stroke Development and Gout

Studies have reported an association between gout and stroke. In 2013, a large population-based cohort study conducted in England concluded that gout was associated with increased risk of all stroke [12]. Reaching a similar conclusion, a retrospective cohort study conducted in the United States in 2017 demonstrated that gout was an incident stroke risk factor equivalent to DM [13]. Compared with patients with DM only, patients with both gout and DM had higher hazard ratios for incident stroke [13]. A population-based study conducted in the United Kingdom also indicated that gout was independently associated with higher risk of AF—a risk factor for stroke—at diagnosis and that the risk of AF was higher after the diagnosis [14]. Another reason gout is associated with increased stroke risk is the pathophysiological effects of a high level of SUA, which may induce an increase in blood pressure [15,16], endothelial inflammation and dysfunction [17,18], impaired nitric oxide production [19,20], and atherosclerosis [21,22,23].

### 4.3. Association between Stroke Risk and Influenza Vaccination

Early research revealed that influenza vaccination may be associated with reduced stroke risk [24]. In 2017, a meta-analysis also indicated that vaccination against influenza was associated with a lower risk of stroke [8]. The present study focused on the effect of influenza vaccination in reducing the stroke risk in people with gout. Our findings revealed that people with gout who were vaccinated had a lower risk of all stroke than those who were unvaccinated. Recent population-based cohort studies using the NHIRD have reached similar conclusions; they have reported that influenza vaccination may reduce the risks of hemorrhagic stroke and ischemic stroke in people with AF [10,11], although the reasons for this finding remain unclear. The possible pathophysiological mechanisms may be related to protection from influenza infection. Influenza infection may cause inflammation, smooth muscle cell proliferation, fibrin deposition in atherosclerotic plaque, and loss of anti-inflammatory properties in high-density lipoproteins [9,25]. These changes may contribute considerably to the development of stroke. A nationwide case–control study conducted in Taiwan also demonstrated that influenza infection was significantly associated with the development of AF, where the AF risk was found to be increased by 18% [26]. Increased risk of stroke for the aforementioned reasons may be reduced by influenza vaccination. Future research should focus on the mechanisms through which influenza vaccination protects against stroke development in people with gout.

### 4.4. Different Postvaccination Stroke Risk Depending on Age and Sex

The most prevalent subtype of hemorrhagic stroke is intracranial hemorrhage (ICH). A systemic review concluded that age was a risk factor for ICH; elderly people had a higher risk of ICH, and this risk approximately doubled with each decade of life [27]. However, the influence of sex on hemorrhagic stroke remains controversial. A study suggested that the risk of ICH before 65 years was generally lower in women than in men, but that this disparity may disappear after the age of 64 years [28]. Conversely, another study reported no significant difference in incident ICH events between the sexes [29]. In our study, people with gout aged >75 years who had been vaccinated did not exhibit a reduced risk of hemorrhagic stroke. Women were not observed to have a lower risk of hemorrhagic stroke after receiving vaccination (Table 3). Through sensitivity analysis, we discovered a lower risk of hemorrhagic stroke in women with gout after they had received ≥4 influenza vaccinations. Regarding ischemic stroke, older age and male sex are risk factors [28]. Both male patients and patients aged >75 years had a lower risk of ischemic stroke after receiving influenza vaccination. We therefore recommend that people with gout who are male or older than 75 years receive influenza vaccination to help prevent ischemic stroke.

### 4.5. Effect of Medications on Stroke Risk

A retrospective cohort study conducted using the NHIRD reported lower risks of hospitalized stroke and all-cause mortality in urate-lowering medication users than in nonusers among patients with gout [30]. In our study, people with gout who used a urate-lowering agent (allopurinol or benzbromarone) for >28 days had significantly lower risks of hemorrhagic and ischemic strokes, indicating that urate-lowering agents and influenza vaccination have an addictive effect on protection from stroke.

Another important medication for stroke risk is aspirin, which has been well reported for stroke primary prevention before [31]. To clarify the possible effect of aspirin on ischemic stroke prevention in the present study, we conduct a subgroup analysis for the sensitivity analysis. The results revealed a potential neuroprotective effect among patients with aspirin usage more or less than 28 days after receiving more than two vaccinations. (Table 4) Therefore, although aspirin may be important for ischemic stroke prevention in the present selected cohort, the potential effect of stoke prevention from influenza vaccination was still observed.

### 4.6. Limitations

The present study had several limitations. First, the NHIRD does not contain data regarding the SUA level, blood sugar, lipid profile, body mass index, or smoking status. Second, we enrolled patients aged >55 years because of the free influenza vaccination policy. Future study is required to investigate younger patients with gout. Third, healthy user bias may have been present [32], although we did adjust for sociodemographic characteristics such as urbanization and monthly income as potential confounders. Fourth, the data were collected until the end of 2012; future studies enrolling more recent data are required to validate our results. Finally, although we controlled for and minimized bias by using propensity score analysis and sensitivity analysis, bias from the uneven prevalence of comorbidities between vaccinated and unvaccinated groups, residual unmeasured confounders, and healthy user bias may still have been present.

## 5. Conclusions

Influenza vaccination was associated with a lower risk of all stroke in patients with gout, and this association appeared to be dose-dependent. Further research is required to determine the potential mechanisms through which influenza vaccination protects against stroke in this patient population.

## Figures and Tables

**Table 1:** Characteristic of the sample population.

	Whole Cohort (n = 23,949)		Unvaccinated (n = 12,300)		Vaccinated (n = 11,649)		*p* ^a^
	n	%	n	%	n	%	
Age, years (Mean ± SD)	66.75 (8.36)		64.22 (8.39)		69.42 (7.44)		<0.001
55–64	11,658	48.68	7973	64.82	3685	31.63	<0.001
65–74	7982	33.33	2711	22.04	5271	45.25	
≥75	4309	17.99	1616	13.14	2693	23.12	
Gender							
Female	10,225	42.69	5194	42.23	5031	43.19	0.133
Male	13,724	57.31	7106	57.77	6618	56.81	
CCI							
0	7243	30.24	3784	30.76	3459	29.69	0.301
1	6328	26.42	3215	26.14	3113	26.72	
2	4498	18.78	2311	18.79	2187	18.77	
≥3	5880	24.55	2990	24.31	2890	24.81	
Comorbidities							
Diabetes	6575	27.45	3414	27.76	3161	27.14	0.282
Hypertension	14,888	62.17	7259	59.02	7629	65.49	<0.001
Dyslipidemia	8985	37.52	4855	39.47	4130	35.45	<0.001
Atrial fibrillation	1891	7.90	748	6.08	1143	9.81	<0.001
Allopurinol							
<28 days	17,859	74.57	9766	79.40	8093	69.47	<0.001
≥28 days	6090	25.43	2534	20.60	3556	30.53	
Benzbromarone							
<28 days	12,633	52.75	7175	58.33	5458	46.85	<0.001
≥28 days	11,316	47.25	5125	41.67	6191	53.15	
Colchicine							
<28 days	16,113	67.28	8621	70.09	7492	64.31	<0.001
≥28 days	7836	32.72	3679	29.91	4157	35.69	
Aspirin							
<28 days	13,284	55.47	7937	64.53	5347	45.90	<0.001
≥28 days	10,665	44.53	4363	35.47	6302	54.10	
Statin							
<28 days	14,272	59.59	7758	63.07	6514	55.92	<0.001
≥28 days	9677	40.41	4542	36.93	5135	44.08	
RAA							
<28 days	9517	39.74	5972	48.55	3545	30.43	<0.001
≥28 days	14,432	60.26	6328	51.45	8104	69.57	
Metformin							
<28 days	18,063	75.42	9543	77.59	8520	73.14	<0.001
≥28 days	5886	24.58	2757	22.41	3129	26.86	
Level of Urbanization							
Urban	16,573	69.20	9159	74.46	7414	63.64	<0.001
Suburban	4713	19.68	2155	17.52	2558	21.96	
Rural	2663	11.12	986	8.02	1677	14.40	
Monthly income (NT$)							
0	2138	8.93	912	7.41	1226	10.52	<0.001
1–21,000	6642	27.73	2925	23.78	3717	31.91	
21,000–33,300	7593	31.70	3359	27.31	4234	36.35	
≥33,301	7576	31.63	5104	41.50	2472	21.22	

^a^ Comparison between the unvaccinated and vaccinated.

**Table 2:** Risk of all stroke among the unvaccinated and vaccinated groups in study cohort.

All Group (n = 23,949)	Unvaccinated (Total Follow-Up 65,692.6 Person-Years)			Vaccinated (Total Follow-Up 92,060.5 Person-Years)			Adjusted HR † (95% C.I.)
	No. of Patients with Stroke	Incidence Rate (per 10^5^ Person-Years) (95% C.I.)		No. of Patients with Stroke	Incidence Rate (per 10^5^ Person-Years) (95% C.I.)		
**Whole cohort**							
All stroke	1500	2283.4	(2167.8, 2398.9)	2084	2263.7	(2166.5, 2360.9)	0.59 (0.55, 0.63) ***
Hemorrhagic stroke	194	295.3	(253.8, 336.9)	241	261.8	(228.7, 294.8)	0.60 (0.49, 0.73) ***
Ischemic stroke	1014	1543.6	(1448.5, 1638.6)	1473	1600.0	(1518.3, 1681.7)	0.60 (0.55, 0.65) ***
**Age, 55–64 ^a^**							
All stroke	757	1652.7	(1534.9, 1770.4)	430	1273.9	(1153.5, 1394.3)	0.54 (0.48, 0.61) ***
Hemorrhagic stroke	119	259.8	(213.1, 306.5)	66	195.5	(148.4, 242.7)	0.53 (0.39, 0.72) ***
Ischemic stroke	495	1080.7	(985.5, 1175.9)	282	835.4	(737.9, 932.9)	0.53 (0.46, 0.62) ***
**Age, 65–74 ^b^**							
All stroke	418	3133.0	(2832.6, 3433.3)	1061	2568.4	(2413.8, 2722.9)	0.63 (0.56, 0.71) ***
Hemorrhagic stroke	42	314.8	(219.6, 410.0)	108	261.4	(212.1, 310.7)	0.64 (0.45, 0.92) *
Ischemic stroke	300	2248.5	(1994.1, 2503.0)	764	1849.4	(1718.3, 1980.9)	0.63 (0.55, 0.72) ***
**Age, ≥75 ^c^**							
All stroke	325	4965.0	(4425.2, 5504.8)	593	3489.1	(3208.3, 3769.9)	0.57 (0.50, 0.65) ***
Hemorrhagic stroke	33	504.1	(332.1, 676.1)	67	394.2	(299.8, 488.6)	0.67 (0.44, 1.02)
Ischemic stroke	219	3345.7	(2902.5, 3788.8)	427	2512.4	(2274.4, 2750.7)	0.60 (0.51, 0.71) ***
**Female ^d^**							
All stroke	571	2092.0	(1920.4, 2263.6)	838	2104.2	(1961.7, 2246.6)	0.62 (0.55, 0.69) ***
Hemorrhagic stroke	57	208.8	(154.6, 263.0)	94	236.0	(188.3, 283.7)	0.76 (0.54, 1.08)
Ischemic stroke	404	1480.1	(1335.8, 1624.5)	587	1473.9	(1354.7, 1593.2)	0.60 (0.52, 0.68) ***
**Male ^e^**							
All stroke	929	2419.4	(2263.8, 2575.0)	1246	2385.4	(2252.9, 2517.8)	0.57 (0.52, 0.63) ***
Hemorrhagic stroke	137	356.8	(297.0, 416.5)	147	281.7	(235.9, 326.)	0.54 (0.42, 0.69) ***
Ischemic stroke	610	1588.6	(1462.6, 1714.7)	886	1696.2	(1584.5, 1807.9)	0.60 (0.53, 0.67) ***

^a^ Total follow-up 45,804.8 person-years for the unvaccinated and 33,754.7 for the vaccinated groups. ^b^ Total follow-up 13,342.0 person-years for the unvaccinated and 41,310.0 for the vaccinated groups. ^c^ Total follow-up 6545.8 person-years for the unvaccinated and 16,995.7 for the vaccinated groups. ^d^ Total follow-up 27,294.6 person-years for the unvaccinated and 39,825.5 for the vaccinated groups. ^e^ Total follow-up 38,398.0 person-years for the unvaccinated and 52,235.1 for the vaccinated groups. C.I.: confidence interval. HR: hazard ratio. † Main model is adjusted for age, sex, Charlson comorbidity index, diabetes, hypertension, dyslipidemia, AF, level of urbanization, and monthly income in propensity score. *: *p* < 0.05, ***: *p* < 0.001.

**Table 3:** Sensitivity analysis of adjusted HRs of vaccination in risk reduction of hemorrhagic stroke in all season.

	Unvaccinated	Vaccinated			*p* for Trend
		1	2–3	≥4	
	Adjusted HR (95%C.I.)	Adjusted HR (95%C.I.)	Adjusted HR (95%C.I.)	Adjusted HR (95%C.I.)	
**Main model** †	1.00	0.81 (0.61, 1.08)	0.80 (0.62, 1.02)	0.37 (0.28, 0.48) ***	<0.001
**Additional covariates** ‡					
Main model + Allopurinol	1.00	0.82 (0.62, 1.09)	0.80 (0.63, 1.03)	0.37 (0.28, 0.49) ***	<0.001
Main model + Benzbromarone	1.00	0.83 (0.62, 1.10)	0.81 (0.63, 1.04)	0.38 (0.29, 0.50) ***	<0.001
Main model + Colchicine	1.00	0.81 (0.61, 1.08)	0.80 (0.62, 1.02)	0.37 (0.28, 0.49) ***	<0.001
Main model + Aspirin	1.00	0.82 (0.62, 1.10)	0.82 (0.63, 1.05)	0.38 (0.29, 0.50) ***	<0.001
**Subgroup effects**					
Age, years					
55–64	1.00	0.51 (0.31, 0.84) **	0.70 (0.47, 1.05)	0.38 (0.23, 0.64) ***	<0.001
≥65	1.00	1.07 (0.74, 1.54)	0.89 (0.64, 1.24)	0.39 (0.28, 0.55) ***	<0.001
Sex					
Female	1.00	1.10 (0.69, 1.74)	0.96 (0.63, 1.46)	0.43 (0.27, 0.69) ***	<0.001
Male	1.00	0.70 (0.49, 1.00)	0.74 (0.54, 1.01)	0.34 (0.24, 0.49) ***	<0.001
Diabetes					
No	1.00	0.75 (0.53, 1.05)	0.76 (0.57, 1.02)	0.37 (0.27, 0.51) ***	<0.001
Yes	1.00	0.99 (0.59, 1.68)	0.87 (0.54, 1.40)	0.33 (0.19, 0.58) ***	<0.001
Dyslipidemia					
No	1.00	0.89 (0.64, 1.23)	0.74 (0.55, 1.00)	0.41 (0.30, 0.56) ***	<0.001
Yes	1.00	0.66 (0.37, 1.18)	0.97 (0.62, 1.53)	0.28 (0.16, 0.51) ***	<0.001
Hypertension					
No	1.00	0.65 (0.40, 1.04)	0.65 (0.42, 0.99) *	0.38 (0.25, 0.60) ***	<0.001
Yes	1.00	0.94 (0.66, 1.34)	0.89 (0.65, 1.22)	0.36 (0.25, 0.51) ***	<0.001
Atrial fibrillation					
No	1.00	0.81 (0.60, 1.09)	0.76 (0.58, 0.99) *	0.35 (0.26, 0.47) ***	<0.001
Yes	1.00	0.93 (0.35, 2.51)	1.27 (0.57, 2.84)	0.59 (0.25, 1.38)	0.299
Allopurinol					
<28 days	1.00	0.74 (0.52, 1.04)	0.84 (0.63, 1.12)	0.33 (0.24, 0.46) ***	<0.001
≥28 days	1.00	1.09 (0.65, 1.84)	0.76 (0.46, 1.26)	0.50 (0.31, 0.82) **	0.003
Benzbromarone					
<28 days	1.00	0.89 (0.61, 1.29)	0.77 (0.54, 1.09)	0.46 (0.32, 0.66) ***	<0.001
≥28 days	1.00	0.75 (0.49, 1.15)	0.86 (0.60, 1.24)	0.31 (0.20, 0.47) ***	<0.001
Colchicine					
<28 days	1.00	0.69 (0.47, 1.01)	0.73 (0.52, 1.00)	0.33 (0.23, 0.47) ***	<0.001
≥28 days	1.00	1.04 (0.68, 1.61)	0.93 (0.62, 1.38)	0.44 (0.28, 0.68) ***	<0.001
Aspirin					
<28 days	1.00	0.75 (0.51, 1.10)	0.74 (0.52, 1.05)	0.32 (0.21, 0.49) ***	<0.001
≥28 days	1.00	0.95 (0.62, 1.45)	0.93 (0.64, 1.34)	0.45 (0.31, 0.67) ***	<0.001
Statin					
<28 days	1.00	0.71 (0.49, 1.01)	0.76 (0.56, 1.03)	0.38 (0.27, 0.54) ***	<0.001
≥28 days	1.00	1.10 (0.69, 1.75)	0.93 (0.60, 1.44)	0.39 (0.24, 0.63) ***	<0.001
RAA					
<28 days	1.00	0.65 (0.36, 1.15)	0.70 (0.42, 1.16)	0.40 (0.23, 0.71) **	0.002
≥28 days	1.00	0.86 (0.62, 1.19)	0.81 (0.61, 1.09)	0.35 (0.26, 0.48) ***	<0.001
Metformin					
<28 days	1.00	0.70 (0.50, 0.98) *	0.74 (0.55, 0.99) *	0.36 (0.26, 0.50) ***	<0.001
≥28 days	1.00	1.29 (0.75, 2.23)	1.04 (0.63, 1.74)	0.42 (0.24, 0.73) **	0.003

*: *p* < 0.05 **: *p* < 0.01 ***: *p* < 0.001. HR: hazard ratio. † Main model is adjusted for age, sex, Charlson comorbidity index, diabetes, hypertension, dyslipidemia, AF, level of urbanization, Monthly income in propensity score. ‡ The models were adjusted for covariates in the main model as well as each additional listed covariate.

**Table 4:** Sensitivity analysis of adjusted HRs of vaccination in the risk reduction of ischemic stroke in all season.

	Unvaccinated	Vaccinated			*p* for Trend
		1	2–3	≥4	
	Adjusted HR (95%C.I.)	Adjusted HR (95%C.I.)	Adjusted HR (95%C.I.)	Adjusted HR (95%C.I.)	
**Main model** †	1.00	0.83 (0.74, 0.94) **	0.73 (0.65, 0.81) ***	0.42 (0.38, 0.47) ***	<0.001
**Additional covariates** ‡					
Main model + Allopurinol	1.00	0.83 (0.74, 0.94) **	0.73 (0.65, 0.81) ***	0.42 (0.38, 0.47) ***	<0.001
Main model + Benzbromarone	1.00	0.84 (0.74, 0.94) **	0.73 (0.66, 0.82) ***	0.43 (0.38, 0.47) ***	<0.001
Main model + Colchicine	1.00	0.83 (0.74, 0.94) **	0.73 (0.65, 0.81) ***	0.42 (0.38, 0.47) ***	<0.001
Main model + Aspirin	1.00	0.78 (0.70, 0.88) ***	0.66 (0.59, 0.73) ***	0.37 (0.34, 0.42) ***	<0.001
**Subgroup effects**					
Age, years					
55–64	1.00	0.60 (0.48, 0.75) ***	0.65 (0.53, 0.80) ***	0.37 (0.29, 0.48) ***	<0.001
≥65	1.00	0.94 (0.82, 1.09)	0.76 (0.67, 0.87) ***	0.43 (0.38, 0.49) ***	<0.001
Sex					
Female	1.00	0.90 (0.75, 1.08)	0.67 (0.56, 0.79) ***	0.43 (0.37, 0.51) ***	<0.001
Male	1.00	0.78 (0.67, 0.92) **	0.77 (0.67, 0.89) ***	0.41 (0.36, 0.48) ***	<0.001
Diabetes					
No	1.00	0.79 (0.68, 0.91) **	0.69 (0.60, 0.79) ***	0.43 (0.38, 0.49) ***	<0.001
Yes	1.00	0.92 (0.75, 1.13)	0.80 (0.66, 0.96) *	0.38 (0.31, 0.46) ***	<0.001
Dyslipidemia					
No	1.00	0.85 (0.73, 0.98) *	0.69 (0.60, 0.79) ***	0.45 (0.40, 0.52) ***	<0.001
Yes	1.00	0.78 (0.64, 0.96) *	0.80 (0.66, 0.96) *	0.34 (0.28, 0.42) ***	<0.001
Hypertension					
No	1.00	0.78 (0.62, 0.97) *	0.68 (0.56, 0.84) ***	0.50 (0.41, 0.61) ***	<0.001
Yes	1.00	0.85 (0.74, 0.98) *	0.72 (0.64, 0.82) ***	0.37 (0.33, 0.42) ***	<0.001
Atrial fibrillation					
No	1.00	0.85 (0.75, 0.97) *	0.77 (0.69, 0.87) ***	0.42 (0.38, 0.48) ***	<0.001
Yes	1.00	0.69 (0.51, 0.93) *	0.52 (0.39, 0.69) ***	0.41 (0.32, 0.54) ***	<0.001
Allopurinol					
<28 days	1.00	0.89 (0.77, 1.03)	0.75 (0.65, 0.85) ***	0.43 (0.38, 0.49) ***	<0.001
≥28 days	1.00	0.72 (0.58, 0.90) **	0.70 (0.58, 0.85) ***	0.42 (0.35, 0.50) ***	<0.001
Benzbromarone					
<28 days	1.00	0.86 (0.73, 1.02)	0.78 (0.67, 0.90) **	0.41 (0.35, 0.48) ***	<0.001
≥28 days	1.00	0.80 (0.67, 0.95) **	0.70 (0.60, 0.82) ***	0.45 (0.39, 0.52) ***	<0.001
Colchicine					
<28 days	1.00	0.89 (0.77, 1.04)	0.76 (0.67, 0.88) ***	0.40 (0.35, 0.46) ***	<0.001
≥28 days	1.00	0.74 (0.60, 0.90) **	0.67 (0.56, 0.81) ***	0.46 (0.39, 0.55) ***	<0.001
Aspirin					
<28 days	1.00	0.90 (0.71, 1.13)	0.68 (0.54, 0.86) ***	0.35 (0.27, 0.45) ***	<0.001
≥28 days	1.00	0.76 (0.66, 0.87) ***	0.66 (0.59, 0.75) ***	0.40 (0.35, 0.45) ***	<0.001
Statin					
<28 days	1.00	0.86 (0.73, 1.01)	0.77 (0.67, 0.90) **	0.45 (0.38, 0.52) ***	<0.001
≥28 days	1.00	0.78 (0.66, 0.93) **	0.68 (0.58, 0.80) ***	0.41 (0.35, 0.48) ***	<0.001
RAA					
<28 days	1.00	0.94 (0.75, 1.19)	0.79 (0.64, 0.99) *	0.46 (0.36, 0.58) ***	<0.001
≥28 days	1.00	0.78 (0.68, 0.90) **	0.70 (0.62, 0.79) ***	0.41 (0.36, 0.46) ***	<0.001
Metformin					
<28 days	1.00	0.78 (0.68, 0.90) ***	0.70 (0.62, 0.80) ***	0.43 (0.38, 0.49) ***	<0.001
≥28 days	1.00	0.95 (0.77, 1.17)	0.78 (0.65, 0.95) *	0.40 (0.33, 0.49) ***	<0.001

*: *p* < 0.05 **: *p* < 0.01 ***: *p* < 0.001. HR: hazard ratio. +CCI Index: Charlson comorbidity index. † Main model is adjusted for age, sex, Charlson comorbidity index, diabetes, hypertension, dyslipidemia, AF, level of urbanization, and monthly income in propensity score. ‡ The models were adjusted for covariates in the main model and each additional listed covariate.

## Data Availability

The data supporting the findings of the present research were sourced from NHIRD in Taiwan. Owing to the legal restrictions imposed by the Government of Taiwan related to the Personal Information Protection Act, the database cannot be made publicly available.
